# RatUNet: residual U-Net based on attention mechanism for image denoising

**DOI:** 10.7717/peerj-cs.970

**Published:** 2022-05-10

**Authors:** Huibin Zhang, Qiusheng Lian, Jianmin Zhao, Yining Wang, Yuchi Yang, Suqin Feng

**Affiliations:** 1 Institute of Information Science and Technology, Yanshan University, Qinhuang Dao, Hebei Province, China; 2 Computer Department, Xinzhou Teachers University, Xinzhou, Shanxi Province, China; 3 Hebei Key Laboratory of Information Transmission and Signal Processing, Yanshan University, Qin Huangdao, Hebei Province, China; 4 School of Information Engineering, Inner Mongolia University of Science and Technology, Baotou, Inner Mongolia Province, China

**Keywords:** Image denoising, Convolutional neural networks, U-Net, Attention mechanism, RatUNet

## Abstract

Deep convolutional neural networks (CNNs) have been very successful in image denoising. However, with the growth of the depth of plain networks, CNNs may result in performance degradation. The lack of network depth leads to the limited ability of the network to extract image features and difficults to fuse the shallow image features into the deep image information. In this work, we propose an improved deep convolutional U-Net framework (RatUNet) for image denoising. RatUNet improves Unet as follows: (1) RatUNet uses the residual blocks of ResNet to deepen the network depth, so as to avoid the network performance saturation. (2) RatUNet improves the down-sampling method, which is conducive to extracting image features. (3) RatUNet improves the up-sampling method, which is used to restore image details. (4) RatUNet improves the skip-connection method of the U-Net network, which is used to fuse the shallow feature information into the deep image details, and it is more conducive to restore the clean image. (5) In order to better process the edge information of the image, RatUNet uses depthwise and polarized self-attention mechanism to guide a CNN for image denoising. Extensive experiments show that our RatUNet is more efficient and has better performance than existing state-of-the-art denoising methods, especially in SSIM metrics, the denoising effect of the RatUNet achieves very high performance. Visualization results show that the denoised image by RatUNet is smoother and sharper than other methods.

## Introduction

Image denoising which can be tried to recover a clean image from its noisy image has been a long-standing problem in low-level vision and image processing (He et al., 2018), and it still remains an active research topic. Recently, deep CNNs have shown their superior performance in image denoising. The success of CNNs for image denoising is attributed to its powerful black-box modeling capacity and great advances in network training and design.

Although CNNs have achieved great success in image denoising, there are the following drawbacks: (1) With the growth of the depth of plain CNNs, CNNs may result in performance degradation and the ability of plain CNNs to extract image features is also limited, so the denoised image has a structural similarity to the original image. (2) Some network models cannot fuse the features of shallow layers into the ones of deep layers. (3) Many network models neglect the edge information of the image, so the denoising effect has poor performance in terms of structural similarity. (4) Many network models divide the training image dataset into a large number of small image patches and use large batch size during the training learning, which result in a large amount of computation and a large number of training iterations per epoch, and result in too long training time.

In this work, we tackle these issues by developing a convolutional denoising network RatUNet. First, Our RatUNet framework uses the denoising U-Net architecture Ronneberger, Fischer & Brox (2015), which has a down-sample path to extract the map feature and an up-sample path to recover a clean map. Secondly, we use the residual blocks of the ResNet He et al. (2016) in the down-sample and up-sample stages of the U-Net structure, which allows the network model to have a deeper depth and avoid to performance saturation. Finally, in order to better process the edge information of the image, we use depthwise Howard et al. (2017) and polarized self-attention (Liu et al., 2021) mechanism to guide a CNN for image denoising. In addition, extensive experiments have shown that RatUNet outperforms state-of-the-art denoising methods on multiple datasets, especially in SSIM, which has reached the best performance as we know.

Our main contributions of this work can be briefly summarized as follows:

(1) A deep U-Net architecture for image denoising is proposed in this work. We have improved the skip-connection method of U-Net network to make sure that the U-Net network has better denoising performance, and the use of residual mapping in U-Net networks can make the network deeper achieving performance improvement.
(2) Depthwise attention mechanism is used to enhance image feature information, and it is very useful to handle complex noisy images. The polarized self-attention mechanism is used to obtain the edge information of the image, thus enhancing the expressiveness of the denoising model.
(3) RatUNet requires a relatively small amount of training data, and the value of training batch size is only 4, and the training time is very small compared to other denoising convolutional neural networks.
(4) RatUNet is superior to the state-of-the-arts methods on five benchmark datasets for image denoising.

## Background

### CNNs for image denoising

The advent of CNNs has greatly improved the Gaussian denoising technology. Traditional denoising methods such as total variation (Osher et al., 2005), BM3D (Dabov et al. (2007) and dictionary learning methods (Chatterjee & Milanfar, 2009) cannot achieve state-of-the-arts denoising performance. Earlier network models were not very effective in image denoising before (Burger, Schuler & Harmeling, 2012) a patch-based algorithm learned with a plain multi-layer perceptron. Subsequently, Zhang et al. (2016) proposed a DnCNN method which uses residual learning and batch normalization (Ioffe & Szegedy, 2015) for image denoising. Zhang, Zuo & Zhang (2018) presented a fast and flexible network (FFDNet) which has the ability to remove spatially variant noise. Yin et al. (2020) presented an output entropy model using a radial basis function neural network which is used for dynamic noise reduction. Tai et al. (2017) proposed a densely connected denoising memory network (MemNet) by introducing a memory block to enable memory of the network. Tian, Xu & Zuo (2020) proposed a BRDNet that enhances network learning by increasing the network width and using Batch renormalization.

The encoder-decoder network framework is an end-to-end learning algorithm, and this structure is well suited for image denoising tasks. Mao, Shen & Yang (2016) first used the encoder-decoder network structure (RedNet) for image denoising. Further, Liu et al. (2018b) introduced a multi-level wavelet CNN (MWCNN) which extracts feature information from noisy images by using the wavelet transform within the encoder-decoder network framework.

Actually, the use of residual blocks in U-Net networks is very common in semantic segmentation networks, such as Zhang, Liu & Wang (2018) and Venkatesh et al. (2018). The denoising network we proposed is to integrate the residual block into U-Net, and combines the advantages of these two networks at the same time, which is suitable for image denoising.

### Attention mechanism

The attention mechanism is widely used in visual tasks, and it is used to address the shortcomings of convolution (Andreoli, 2019; Bello et al., 2019; Prajit et al., 2019), and has achieved remarkable success. In recent years, there has been some researches on the use of attention mechanisms for image denoising. Image non-local self-similarity attention mechanism has been an useful prior for the image denoising. There are many network models that use non-local self-similarity, such as UNLNet (Lefkimmiatis, 2018) and N3Net (Roth, 2018). Recently, Liu et al. (2018a) proposed a non-local self-similarity recurrent network (NLRN). Tian et al. (2020) proposed an attention-guided denoising convolutional neural network (ADNet).

These attention mechanisms can be added to CNNs as plug-and-play modules. Howard et al. (2017) proposed MobileNets which is built primarily from depthwise separable convolutions. The depthwise separable convolutions can enhance image feature information, and it is regarded as a depthwise attention mechanism. Liu et al. (2021) presented the polarized self-attention (POSA) block which has high internal resolution in both channel and spatial attention computation. Thus, it is helpful for image denoising.

Inspired by attention mechanisms, we integrate depthwise separable convolutions and POSA block into CNN for image denoising in this article.

## Methods

In this section, we briefly introduce image denoising based on mathematical model for supervised learning. Then, we present our RatUNet architecture.

### CNNs model for image denoising

The mathematical model of noise image and clean image can generally be expressed as follows:

(1) $${I_n} = {I_c} + n$$

where, *I*_*c*_ is a clean image, *n* is Additive White Gaussian Noise (AWGN) with variance *σ*^2^, *I*_*n*_ is a noisy image, and *I*_*n*_, *I*_*c*_, *n* ∈ *R*^*M*×*N*×*C*^, respectively, *M*, *N* represent the width and height of image, *C* is the number of image channels.

The most common method with supervised learning to recover **I**_*c*_ from **I**_*n*_ is least-squares, if *a priori* knowledge is available and there is a the regular term. We set the mathematical expression of this denoiser to be *τ*(**I**_*n*_), and the minimization optimization function is as follows:

(2) $$\widehat I = \mathop {\arg \min }\limits_{{I_c}} \displaystyle{1 \over 2}{\rm \parallel }\tau ({I_n}) - {I_c}{{\rm \parallel }_2} + \lambda R(\tau ({I_n}),{I_c})$$

where the problem to be solved is to minimize the data term $\textstyle{1 \over 2}{\rm \parallel }\tau ({I_n}) - {I_c}{{\rm \parallel }_2}$ and a regularization term (also known as penalty term) *λ R*(*τ*(*I*_*n*_),*I*_*c*_) with regularization parameter *λ*. If there is no regular term, then the Eq. (2) is as follows:

(3) $$\widehat {\bf I} = \mathop {\arg \min }\limits_{{{\bf I}_c}} \displaystyle{1 \over 2}{\rm \parallel }\tau ({{\bf I}_n}) - {{\bf I}_c}{{\rm \parallel }_2}$$

### Architecture of RatUNet

**Figure 1:**
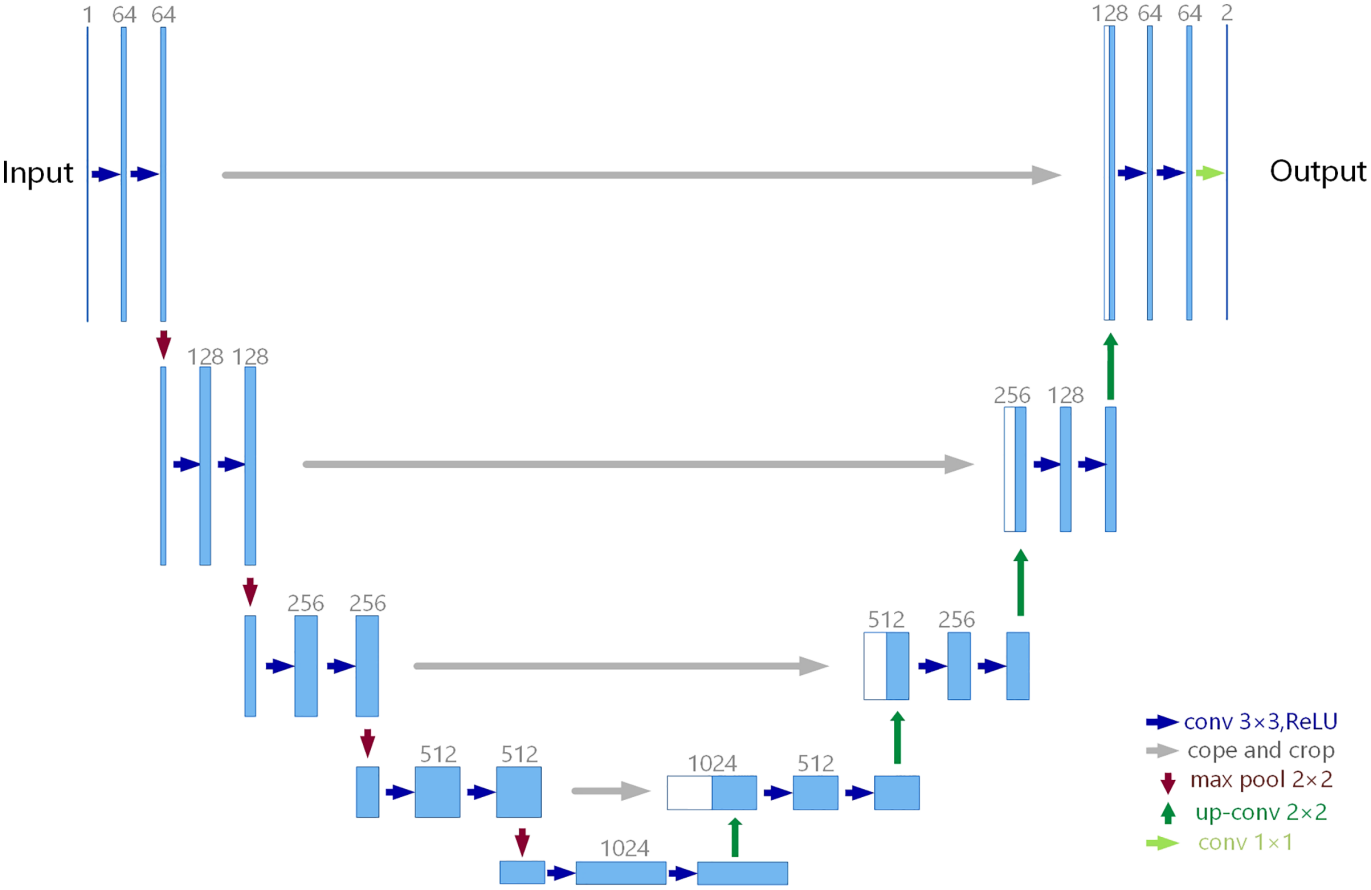
U-Net architecture.

U-Net is a well-known image segmentation network in medical image processing, and its network structure is shown in Fig. 1. The network structure of U-Net is mainly divided into three parts: down-sampling, up-sampling and skip connection. U-Net is Encoder-Decoder structure, the left structure is the down-sampling process, which is the Encoder structure, and the right is the up-sampling process, which is the Decoder structure. Encoder is responsible for feature extraction, in other words, the image size is reduced by down-sampling and convolution to extract some features in shallow layers. Decoder obtains some global features in deep layers through up-sampling and convolution. In the shallow feature map, there is more detailed information (local features); in the deep feature map, there is more contextual information (global features), so the network performance may be better if the features of different deep and shallow layers are fused in multiple scales.

**Figure 2:**
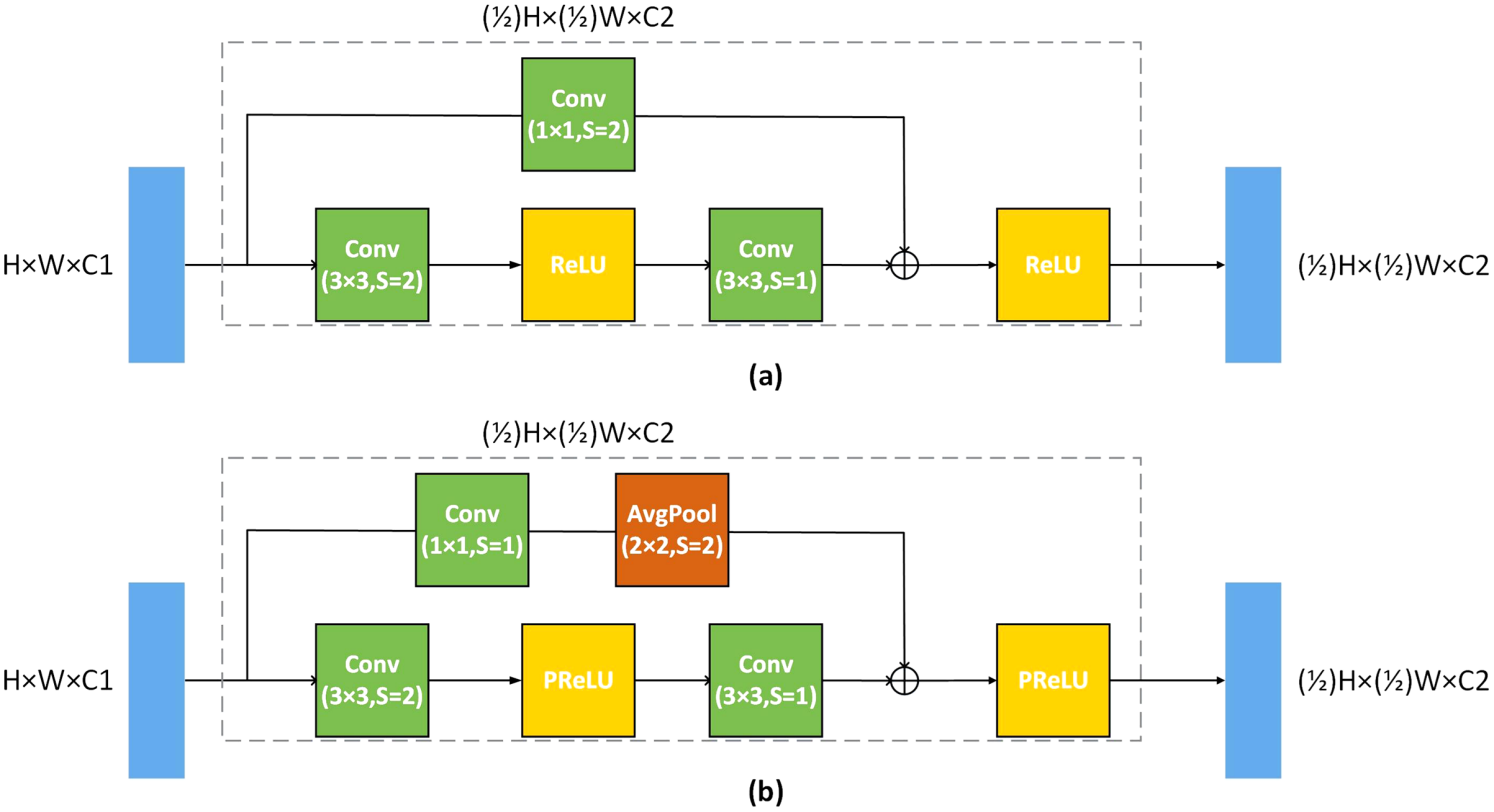
Residual block architecture. (A) The standard residual block of the residual network, (B) the improved residual block, and s is stride.

In order to make the competent for the image denoising task, we have made some improvements to U-Net. A deeper network depth can extract more feature information of the image, but as the plain network depth deepens, the network performance will be saturated. To deepen the depth of the network, convolutional layer of our U-Net is composed of residual blocks. U-Net uses Maxpool with 2 × 2 kernel and two strides to implement down-sampling, and we replace the Maxpool with a 3 × 3 convolution kernel with 2 strides. We use a 1 × 1 convolution kernel with 1 stride and avgpool with 2 × 2 kernel to replace the residual connection of different scales, where the kernel size of avgPool is 2 × 2 He et al. (2019), as shown in Fig. 2. This residual connection method will make more information to flow from the large-scale convolutional layer to the small-scale convolutional layer. Meanwhile, the average pooling is equivalent to the mean filter, which is more conducive to image denoising.

The up-sampling of U-Net generally uses interpolation methods or uses 2 × 2 up-convolution kernel with 2 strides, and we replace it with a 3 × 3 deconvolution kernel with two strides.

We set the output of the encoder with the same size of the feature map on the left side of the U-Net network as *O*_*en*_, and perform a concatenation skip-connection with the up-sampling on the right, and set the concatenation operation to $\oplus$. The convolution layer after the skip-connection is used as the decoder, and the output is set to *O*_*de*_. The decoder for this stage is set to the function *f*_*de*_, and the expression of their relationships as follows:

(4) $${O_{de}} = {f_{de}}(up \oplus {O_{en}})$$

We think that the shallow feature information enters the decoder prematurely, which is not conducive to the decoder to extract the global feature information, so we concatenate the output of the encoder and the output of the decoder. The decoder output of this stage can is expressed as:

(5) $${O_{de}} = {f_{de}}(up) \oplus {O_{en}}$$

**Figure 3:**
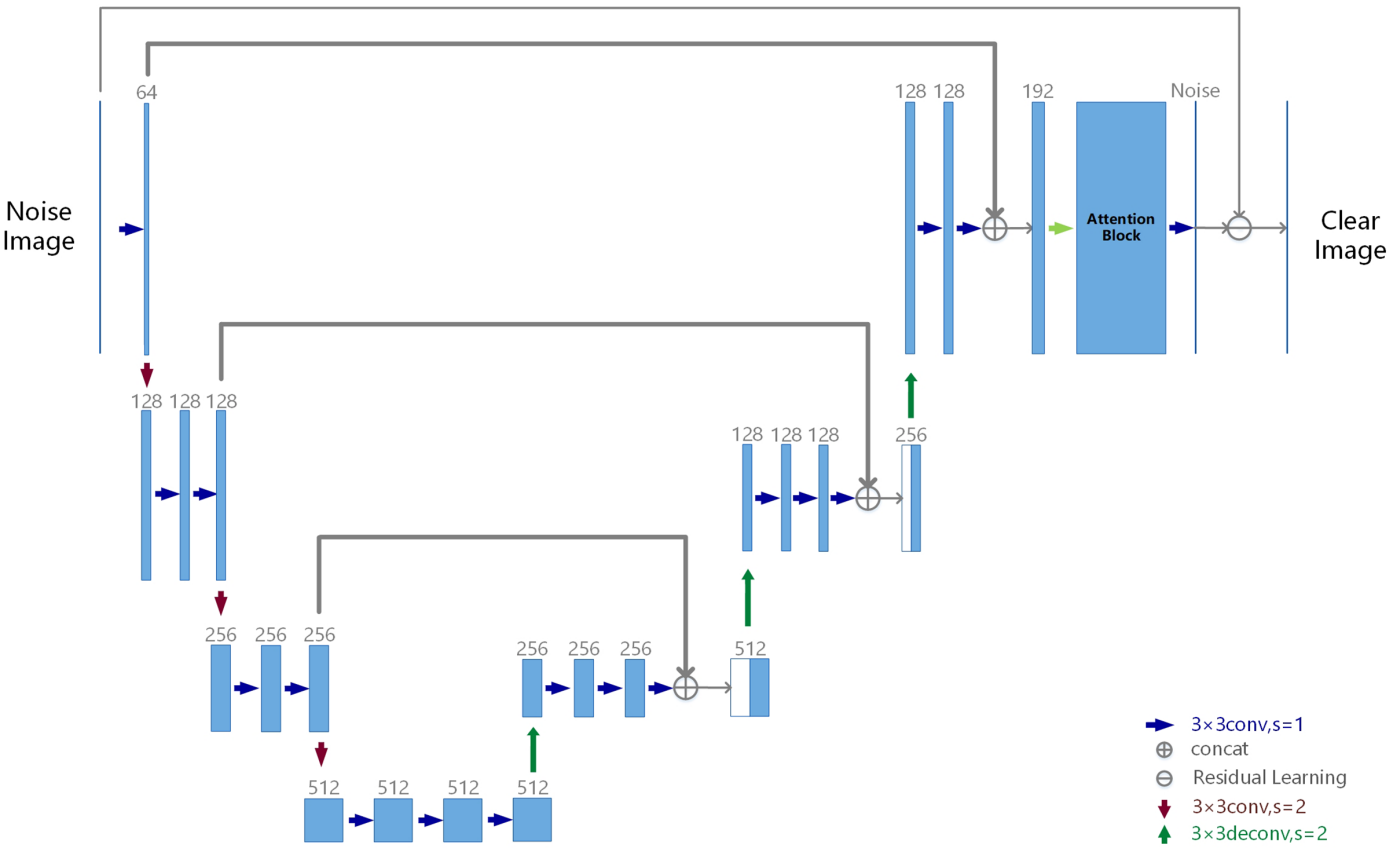
RatUNet architecture. The numbers 128, 256, and 512 in the figure represent channels, and the rectangular block in the figure represents the standard residual blocks which are composed of two conv3 × 3.

Our RatUNet is down-sampled three times and up-sampled twice, respectively. No activation function is used in the first and last convolutional layers, and similarly no activation function is used in the deconvolution (up-convolution) layer. In addition, the activation function uses the PRelu function. The architecture of RatUNet is illustrated in Fig. 3.

### Attention block

**Figure 4:**
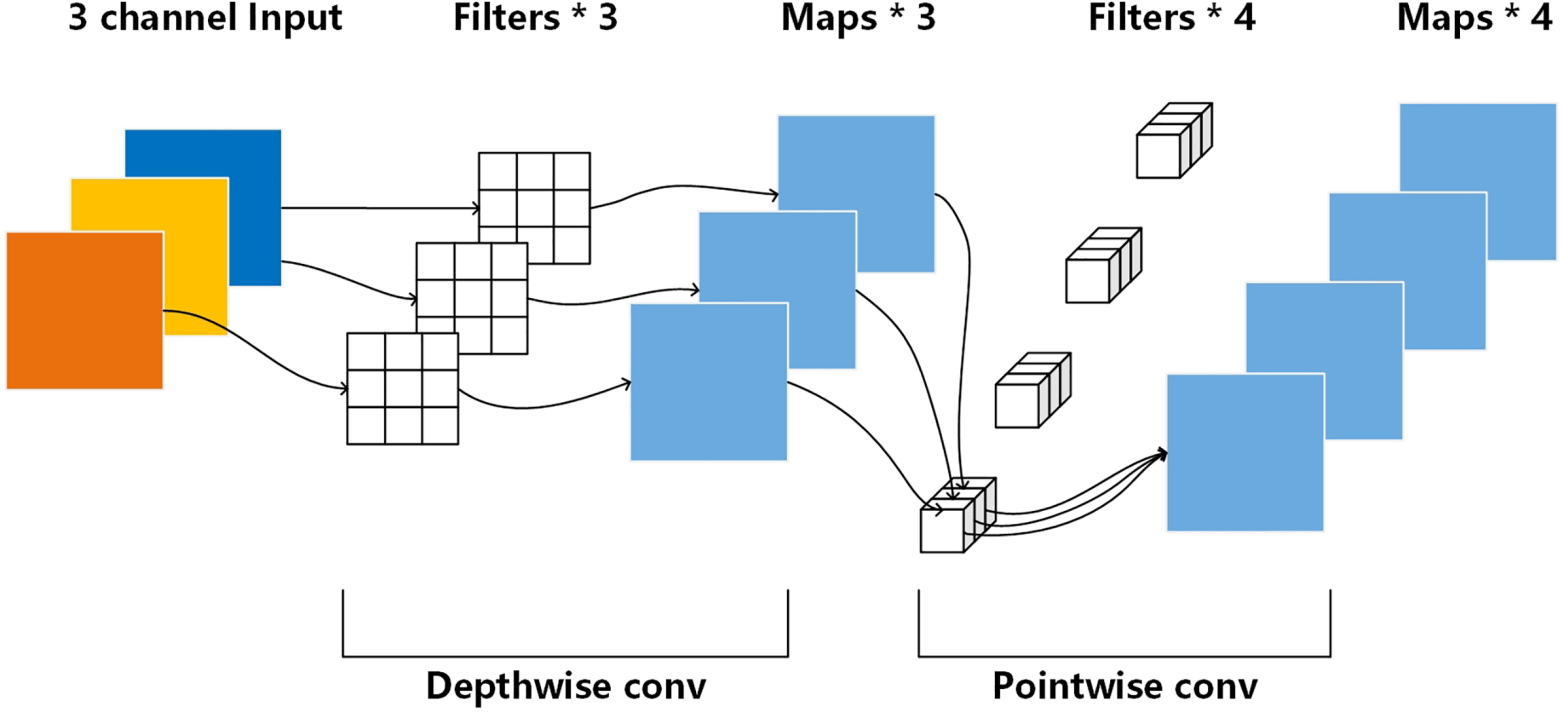
Depthwise attention mechanism (Howard et al., 2017).

The attention block of RatUNet consists of depthwise attention mechanism and polarized self-attention mechanism. Depthwise attention mechanism (Howard et al., 2017) is used to enhance the feature information of each channel, as shown in Fig. 4.

**Figure 5:**
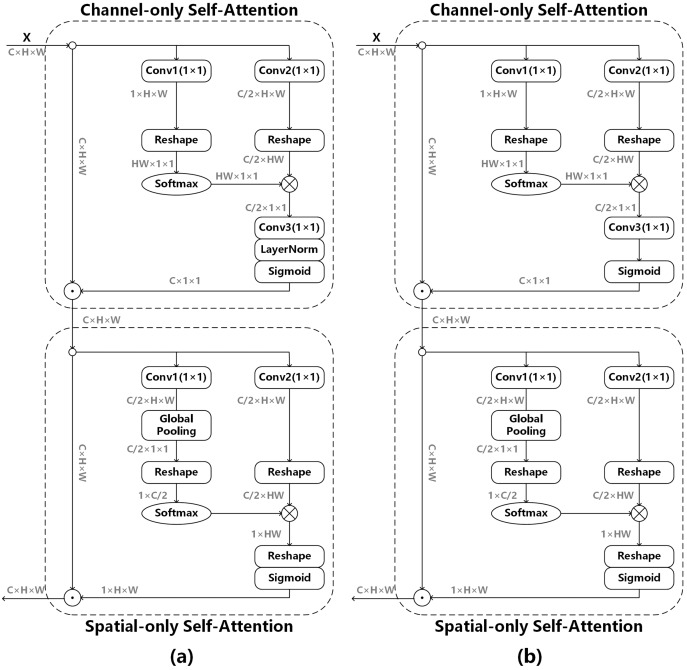
Polarized self-attention mechanism. (A) The original attention mechanism (Liu et al., 2021), (B) the improved attention mechanism after removing LayerNorm.

The polarized self-attention mechanism (Liu et al., 2021) is used to obtain the edge information of the image, as shown in (a) of Fig. 5, and its model can be expressed as follows:

Channel-only branch can be expressed as:

(6) $${O_{ch}}({\bf x}) = Sigmoid(LN[Conv(R1(conv({\bf x})) \times Softmax(R2(Conv({\bf x}))))])$$

Spatial-only branch can be expressed as:

(7) $${O_{sp}}({\bf x}) = Sigmoid(R3[Softmax(R1(GP(Conv({\bf x}))) \times R2(Conv({\bf x})))])$$

where Conv is 1 × 1 convolution operator, *R*1, *R*2 and *R*3 are three tensor reshape operators, GP is a global pooling operator, LN is a LayerNorm operator, and × is the matrix dot-product operation.

We do not use BatchNorm in the whole network model. If LayerNorm is used in the attention mechanism, the data distribution will change greatly, which is not conducive to the adjustment of the subsequent convolutional layers, so LayerNorm is removed from Channel-only branch. Channel-only branch can be expressed as follows:

(8) $${O_{ch}}({\bf x}) = Sigmoid(Conv(R1(conv({\bf x})) \times Softmax(R2(Conv({\bf x})))))$$

The outputs of above two branches are composed either under the sequential layout, and the formula is as follows:

(9) $$POSA({\bf x}) = {O_{sp}}({O_{ch}}{{\rm \odot }^{ch}}{\bf x}){{\rm \odot }^{sp}}{O_{ch}}{{\rm \odot }^{ch}}{\bf x}$$

where ${{\rm \odot }^{ch}}$ is a channel-wise multiplication operator, and ${{\rm \odot }^{sp}}$ is a spatial-wise multiplication operator.

### Loss function

We let the mathematical function of RatUNet be *f*_*RatUNet*_. The objective training is to minimize the mean square error (MSE) (Douillard et al., 2010) to obtain the optimal parameters of RatUNet, and Denote the parameters of RatUNet by *θ*, and *f*_*RatUNet*_(**I**_*n*_; *θ*) is the output of the network. We adopt the Adam algorithm (Kingma & Ba, 2015) to optimize the parameters of RatUNet by minimizing the following objective function (loss function):

(10) $$L(\theta ) = \displaystyle{1 \over {2N}}\sum\limits_{i = 1}^N {\rm \parallel }{f_{RatUNet}}({I_{n,i}};\theta ) - {I_{n,i}} + {I_{c,i}}{{\rm \parallel }_2}$$

where *N* is the number of noisy-image.

## Results

### Training datasets

To train our RatUNet for Additive White Gaussian Noise (AWGN) denoising, our training set is constructed by using images from Berkeley Segmentation Dataset (BSD) (Martin et al., 2001) and DIV2K (Agustsson & Timofte, 2017). Specifically, we collected 400 images from the train and test partitions of BSD and 800 images from DIV2K. In order to increase the diversity of network training samples, we augment the training data set as follows: (1) The 400 BSD images were rotated counterclockwise by 90, 180 and 270 degrees and were flipped horizontally to finally generate 3,200 images. (2) The 800 DIV2K images were flipped horizontally and up-down, resulting in 3,200 images. After the above data augmentation, our training dataset has *N* = 4 × 1,600 images. Image patches with the size of 160 × 160 are randomly cropped from the training image during the training stage.

#### Test datasets

To evaluate the performance of RatUNet for grayscale image denosing, we perform on two datasets *i.e*. Set12 and BSD68 (Roth & Black, 2005). BSD68 is composed of 68 images from test set of the BSD dataset, and its size is 321 × 481. Set12 is composed of 12 images, of which the size of 7 images is 256 × 256 and the size of 5 images is 512 × 512.

For color image denosing, we evaluate the performance of RatUNet on three datasets *i.e*. CBSD68 (Roth & Black, 2005), Kodak24 (Franzen, 1999) and McMaster (Zhang et al., 2011). CBSD68 is a color version corresponding to the grayscale BSD68 dataset. Kodak24 is composed of 24 center-cropped images, and the size of these images is 500 × 500 from the Kodak dataset. McMaster is composed of 18 images of size 500 × 500.

### Implementation details

We used the Adam algorithm with *β*_1_ = 0.9, *β*_2_ = 0.999, and *ε* = 10^−8^ to minimize the loss function for training RatUNet. The batch-size is four and patch size is 160 × 160. The initial learning rate is 0.0001, and by using the cosine learning rate decay method (He et al., 2019) which decays the learning rate after each iteration and decays to 0 after the last iteration. Training epochs is 100, and BatchNorm is not used.

The initialization of the convolution kernel has a great influence on the convergence speed of CNNs and whether it can converge. Now there are many initialization methods for CNNs, such as Xavier initialization method (Glorot & Bengio, 2010) and the GLIT method (Liu, Liu & Zhang, 2022). CNNs are generally initialized using the kaiming initialization method (He et al., 2015). According to the necessary conditions for the convergence of CNNs and the initialization formula of the convolution kernel proposed by Zhang et al. (2022), we initialize the bias to 0 and the convolution kernel weights are initialized as follows:

(11) $${{\bf w}_l}{\rm \sim }N\left(0,\displaystyle{{0.9} \over {{n_l}}}\right)$$

where **w**_*l*_ represents the weight parameter of the convolution kernel of the layer *l*, which is randomly distributed according to the Gaussian function with mean value 0 and variance of $\textstyle{{0.9} \over {{n_l}}}$, and ${n_l} = {c_l} \times k_l^2$. *c*_*l*_ is the number of output channels of layer *l*, and *k*_*l*_ is the size of the convolution kernel.

We use Pytorch version 1.3 and Python version 3.7 to train and test RatUNet, and all experiments run on a PC with NVIDIA GTX 1070ti GPU.

### Comparisons with state-of-the-art methods

In this subsection, we compare the proposed RatUNet with state-of-the-art models for AWGN task of grayscale images and color images denoising.

For grayscale image denoising, the experimental results of the RatUNet are compared with a number of recent state-of-the-art methods, such as BM3D (Dabov et al., 2007), WNNM (Gu et al., 2014), TNRD (Chen & Pock, 2017), DnCNN (Zhang et al., 2016), IRCNN (Zhang et al., 2017), FC-AIDE (Cha & Moon, 2019), NLRN (Liu et al., 2018a), GCDN (Valsesia, Fracastoro & Magli, 2020) and MWCNN (Liu et al., 2018b). Among the compared methods, BM3D, WNNM and TNRD are three representative model-based methods, and the remaining methods are based on CNN denoising methods. SSIM (Wang et al., 2004) and PSNR are used to quantitatively measure the image denoising performance.

As shown in Table 1, it can be seen that the proposed RatUNet achieves the state-of-the-art performance on noise levels of 15, 25 and 50, and outperforms the state-of-the-art denosing method. All results have been provided by the authors’ literature.

**Table 1:** Average PSNR(dB)/SSIM values of the state-of-the-art methods for grayscale image denoising with various noise levels σ = 15, 25 and 50 on benchmarks datasets Set12 and BSD68. Red color indicates the best performance and second best performances are highlighted in blue.

Dataset	Noise	BM3D	WNNM	TNRD	DnCNN	IRCNN	FC-AIDE	NLRN	GCDN	MWCNN	RatUNet
Set12	15	32.37	32.70	32.50	32.86	32.77	32.99	**33.16**	33.14	33.15	**33.16**
		0.8952	0.8982	0.8958	0.9031	0.9008	0.9006	0.9070	0.9072	**0.9088**	**0.9110**
	25	29.97	30.28	30.06	30.44	30.38	30.57	**30.80**	30.78	30.79	**30.85**
		0.8504	0.8557	0.8512	0.8622	0.8601	0.8557	0.8689	0.8687	**0.8711**	**0.8736**
	50	26.72	27.05	26.81	27.18	27.14	27.42	27.64	27.60	**27.74**	**27.76**
		0.7676	0.7775	0.7680	0.7829	0.7804	0.7768	0.7980	0.7957	**0.8056**	**0.8049**
BSD68	15	31.07	31.37	31.42	31.73	31.63	31.78	**31.88**	31.83	31.86	**31.87**
		0.8717	0.8766	0.8769	0.8907	0.8881	0.8907	0.8932	0.8933	**0.8947**	**0.8999**
	25	28.57	28.83	28.92	29.23	29.15	29.31	29.41	29.35	29.41	**29.43**
		0.8013	0.8087	0.8093	0.8278	0.8249	0.8281	0.8331	0.8332	**0.8360**	**0.8419**
	50	25.62	25.87	25.97	26.23	26.19	26.38	26.47	26.38	**26.53**	**26.53**
		0.6864	0.6982	0.6994	0.7189	0.7171	0.7181	0.7298	**0.7389**	0.7366	**0.7399**

In Table 1, we do not list other methods such as ADNet (Tian et al., 2020), FFDNet (Zhang, Zuo & Zhang, 2018), FOCNet (Jia et al., 2019), BRDNet (Tian, Xu & Zuo, 2020), RNAN (Zhang et al., 2019) and RDN (Zhang et al., 2021) for two reasons: first, their average PSNR performances are smaller than RatUNet, and second, these methods do not provide SSIM performances.

**Figure 6:**
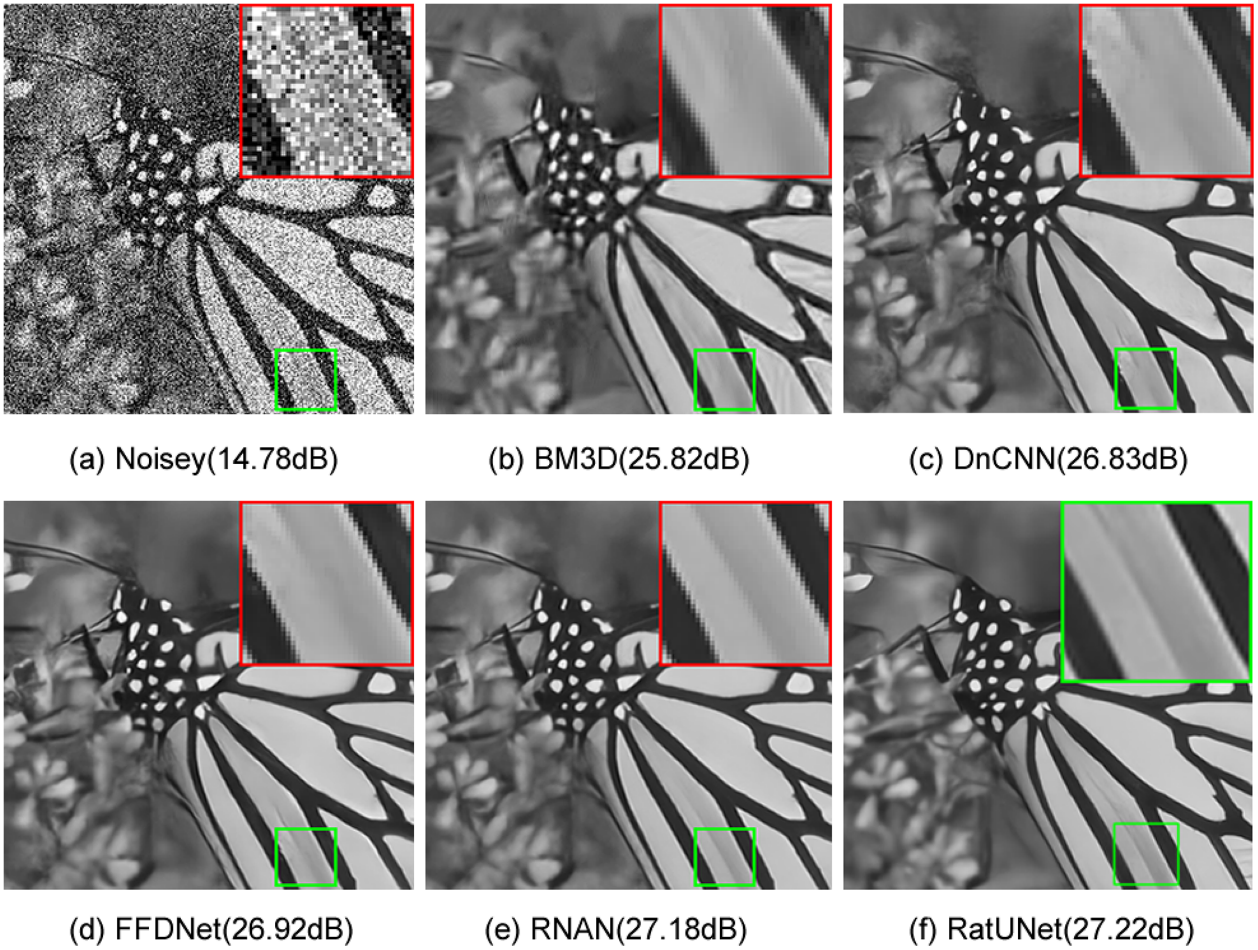
(A–F) Visual comparison results for grayscale image denoising methods on the image “Monarch” from Set12 dataset with noise level 50.

As can also seen Fig. 6, which shows a visual comparison on an image from the Set12 dataset and illustrates the visual results from BM3D, DnCNN, FFDNet and RNAN. Figure 6 shows the results of grayscale image denoising with different methods by noise level 50 on image “102061” from BSD68 dataset. As can be seen from Fig. 6, competitive methods have aliasing and blurring on the edge of the image. One can see that RatUNet produces a much clearer image than other methods and can produce better edge information than the above methods.

We compare RatUNet with CBM3D, DnCNN, IRCNN, ADNet, BRDNet and FFDNet on three benchmark datasets *i.e*. CBSD68, Kodak24 and McMaster for color image denoising. Table 1 reports the color image denoising results of different methods for noise levels 15, 25 and 50. As shown in Table 2, we can see that RatUNet achieves excellent results and outperforms the other methods on different noise levels for color Gaussian noisy image denoising.

**Table 2:** Average PSNR(dB) values of the state-of-the-art methods for color image denoising with various noise levels σ = 15, 25 and 50 on benchmarks datasets CBSD68, Kodak24 and McMaster. Red color indicates the best performance and second best performances are highlighted in blue.

Dataset	Noise	CBM3D	DnCNN	IRCNN	FFDNet	BRDNet	ADNet	RNAN	RatUNet
CBSD68	15	33.52	33.90	39.86	33.87	34.10	33.99	–	**34.20**
	25	30.71	31.24	31.16	31.21	31.43	31.31	–	**31.55**
	50	27.38	27.95	27.86	27.42	28.16	28.04	**28.27**	**28.36**
Kodak24	15	34.28	34.60	34.69	34.63	**34.88**	–	34.76	**35.08**
	25	32.15	32.14	32.18	32.13	32.41	32.26	–	**32.64**
	50	28.46	28.95	28.93	28.98	29.22	29.10	**29.58**	**29.60**
McMaster	15	34.06	33.45	34.58	34.66	**35.08**	34.93	–	**35.10**
	25	31.66	31.52	32.18	32.35	**32.75**	32.56	–	**32.80**
	50	28.51	28.62	28.91	29.18	**29.52**	29.36	–	**29.72**

**Figure 7:**
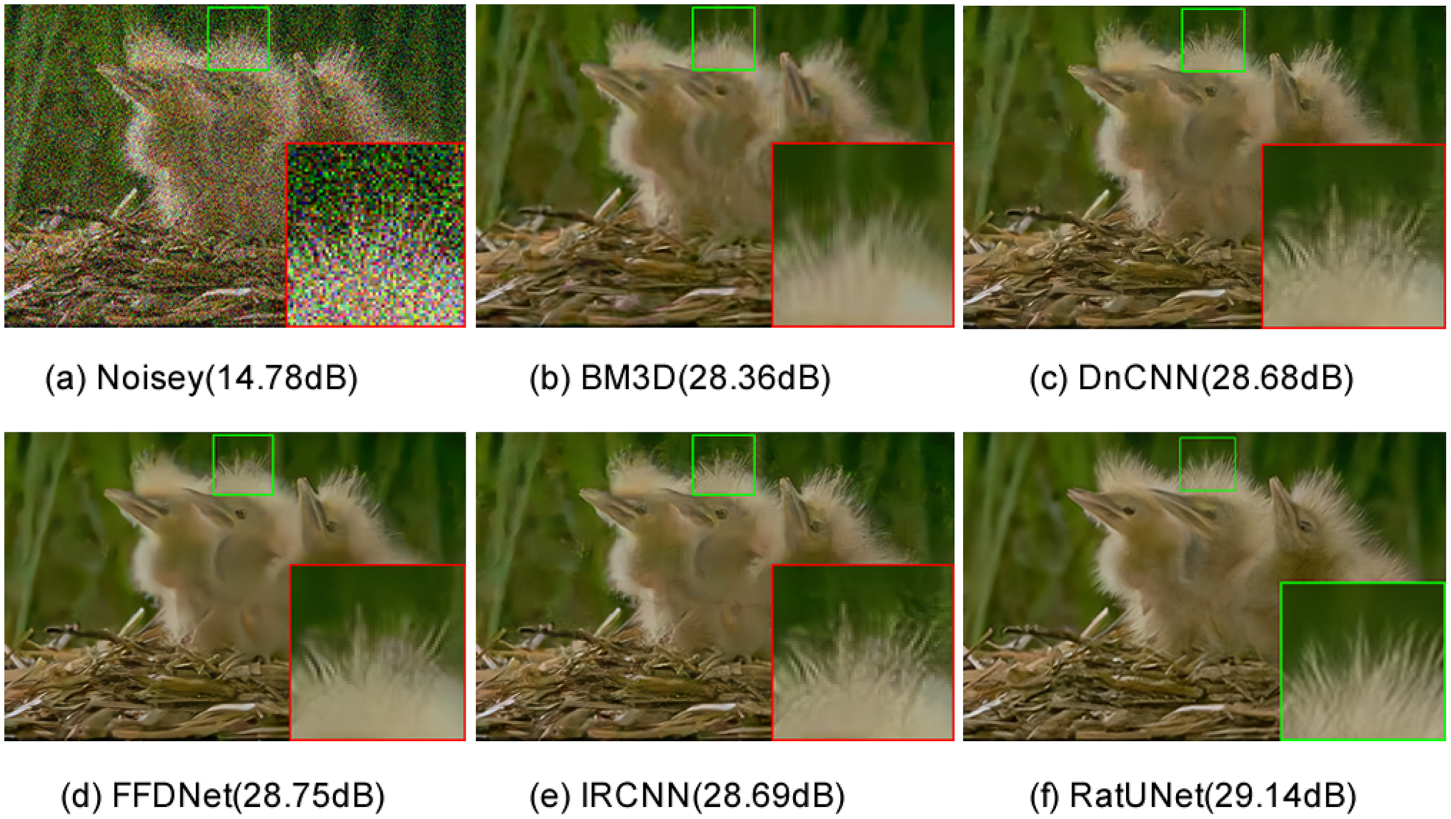
(A–F) Color image denoising results of one image “163085” from the CBSD68 dataset with noise level 50 for different methods.

The visual comparison results on image “163085” by noise level 50 from the CBSD68 dataset with noise level 50 for different methods are shown in Fig. 7. As we can see, RatUNet can remove serious noise and retain the rich edge information of the image, resulting in better edges and more natural textures. Due to the high SSIM value of our method, the denoised image has a high structural similarity to the clean image.

### Training time

Table 3 shows the GPU run training time of the competing methods. Except that DnCNN is our re-implementation of the training of the original model, the training time of other network models are all data provided by the paper. As can be seen from Table 3, although the computing power of our GPU is much smaller than computing power of the GPU used in the above methods, the training time of our RatUNet is much smaller than that of the other methods, and many performances are higher than the above methods.

**Table 3:** The training time comparison with different GPU.

Method	GPU	Stream processor unit	Memory capacity (GB)	Training time (h)
FFDNet	Nvidia Titan X	3,584	12	48
FOCNet	Nvidia Titan Xp	3,840	12	48
NLRN	Nvidia Titan Xp	3,840	12	72
MWCNN	Nvidia GTX 1080	2,560	8	48
DnCNN	Nvidia GTX 1070ti	2,432	8	15
RatUNet	Nvidia GTX 1070ti	2,432	8	11

## Discussion

The appropriate activation function will give the network model better denoising performance. Keeping the other parameters of the network model unchanged, the two activation functions, ReLU and PReLU, are selected for the experiments to test the influence of the activation function on the evaluation index of denoising performance, and the comparison of the experimental results for grayscale image denoising with noise level *σ* = 25 is shown in Table 4. As can be seen from Table 4, the denoising effect of the PReLU activation function is slightly better than that of the ReLU activation function

**Table 4:** Performance comparison of the denoising results of the ReLU and PReLU activation functions of the network model.

Dataset	Performance metrics	ReLU	PReLU
Set12	PSNR	30.81	30.85
	SSIM	0.8730	0.8736
BSD68	PSNR	29.39	29.43
	SSIM	0.8408	0.8419

**Figure 8:**
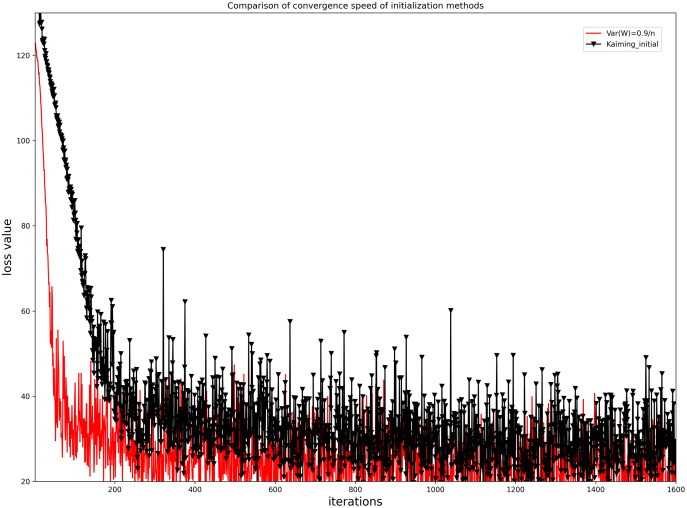
Comparison of the iterative convergence of loss function values for our initialization method and the Kaiming initialization method.

To evaluate the convergence performance of the initialization method of Eq. (11), we compared it with the kaiming initialization method, as shown in Fig. 8. As can be seen from Fig. 8, the initialization method used for our network model converges faster than the kaiming initialization method and the loss function values fluctuate less and are more stable.

## Conclusions

In this work, we propose an residual U-Net framework based on the attention mechanism CNNs as well as RatUNet for image denoising. RatUNet improves the UNet network structure in terms of down-sampling, up-sampling and skip-connection, and introduced the residual block into the U-Net network structure, and at the same time improved the skip-connection method of the residual block. Finally, we use the depthwise and polarized self-attention mechanism to guide the U-Net framework for image denoising. The training time of RatUNet is much less than other network models. The experimental results show that our RatUNet can provide a significant performance gain and is more effective than the state-of-the-art methods for image denoising. In the future, we will focus on extending RatUNet to other vision tasks, such as image deblurring and super-resolution.

## Supplemental Information

10.7717/peerj-cs.970/supp-1 Supplemental Information 1 Code source file.

## Additional Information and Declarations

### Data Availability

The following information was supplied regarding data availability: The raw data used for the experiments are available at: - Train dataset BSD: https://www2.eecs.berkeley.edu/Research/Projects/CS/vision/grouping/resources.html. - Train dataset DIV2K: https://data.vision.ee.ethz.ch/cvl/DIV2K - Test dataset Kodak24: http://r0k.us/graphics/kodak - Test dataset McMaster: https://web.comp.polyu.edu.hk/cslzhang/CDM_Dataset.htm The code file is available in the Supplemental File. The weight parameter files of the experimental run results are available at GitHub: https://github.com/Zhanghb1688/RatUNet.

## References

[ref-1] Agustsson E, Timofte R (2017). Ntire 2017 challenge on single image super-resolution: dataset and study.

[ref-17] Andreoli JM (2019). Convolution, attention and structure embedding.

[ref-2] Bello I, Zoph B, Le Q, Vaswani A, Shlens J (2019). Attention augmented convolutional networks.

[ref-3] Burger HC, Schuler CJ, Harmeling S (2012). Image denoising: can plain neural networks compete with bm3d?.

[ref-4] Cha S, Moon T (2019). Fully convolutional pixel adaptive image denoiser.

[ref-5] Chatterjee P, Milanfar P (2009). Clustering-based denoising with locally learned dictionaries. IEEE Transactions on Image Processing.

[ref-6] Chen Y, Pock T (2017). Trainable nonlinear reaction diffusion: a flexible framework for fast and effective image restoration. IEEE Transactions on Pattern Analysis and Machine Intelligence.

[ref-7] Dabov K, Foi A, Katkovnik V, Egiazarian K (2007). Image denoising by sparse 3-d transform-domain collaborative filtering. IEEE Transactions on Image Processing.

[ref-8] Douillard C, Jézéquel M, Berrou C, Electronique D, Picart A, Didier P, Glavieux A (2010). Iterative correction of intersymbol interference: turbo-equalization. European Transactions on Telecommunications.

[ref-9] Franzen R (1999). Kodak lossless true color image suite: Photocd pcd0992. http://r0k.us/graphics/kodak.

[ref-10] Glorot X, Bengio Y (2010). Understanding the difficulty of training deep feedforward neural networks.

[ref-11] Gu S, Zhang L, Zuo W, Feng X (2014). Weighted nuclear norm minimization with application to image denoising.

[ref-12] He K, Zhang X, Ren S, Sun J (2015). Delving deep into rectifiers: surpassing human-level performance on imagenet classification.

[ref-13] He K, Zhang X, Ren S, Sun J (2016). Deep residual learning for image recognition.

[ref-37] He W, Zhang H, Shen H, Zhang L (2018). Hyperspectral image denoising using local low-rank matrix recovery and global spatial spectral total variation. IEEE Journal of Selected Topics in Applied Earth Observations and Remote Sensing.

[ref-14] He T, Zhang Z, Zhang H, Zhang Z, Xie J, Li M (2019). Bag of tricks for image classification with convolutional neural networks.

[ref-15] Howard AG, Zhu M, Chen B, Kalenichenko D, Wang W, Weyand T, Andreetto M, Adam H (2017). Mobilenets: efficient convolutional neural networks for mobile vision applications.

[ref-16] Ioffe S, Szegedy C (2015). Batch normalization: accelerating deep network training by reducing internal covariate shift.

[ref-18] Jia X, Liu S, Feng X, Zhang L (2019). Focnet: a fractional optimal control network for image denoising.

[ref-19] Kingma D, Ba J (2015). Adam: a method for stochastic optimization.

[ref-20] Lefkimmiatis S (2018). Universal denoising networks: a novel CNN architecture for image denoising.

[ref-21] Liu D, Wen B, Fan Y, Loy CC, Huang TS (2018a). Non-local recurrent network for image restoration.

[ref-22] Liu H, Liu F, Fan X, Huang D (2021). Polarized self-attention: towards high-quality pixel-wise regression. ArXiv preprint.

[ref-23] Liu J, Liu Y, Zhang Q (2022). A weight initialization method based on neural network with asymmetric activation function. Neurocomputing.

[ref-24] Liu P, Zhang H, Zhang K, Lin L, Zuo W (2018b). Multi-level wavelet-CNN for image restoration.

[ref-38] Mao XJ, Shen C, Yang YB (2016). Image restoration using very deep convolutional encoder-decoder networks with symmetric skip connections. ArXiv preprint.

[ref-25] Martin D, Fowlkes C, Tal D, Malik J (2001). A database of human segmented natural images and its application to evaluating segmentation algorithms and measuring ecological statistics.

[ref-26] Osher S, Burger M, Goldfarb D, Xu J, Yin W (2005). An iterative regularization method for total variation-based image restoration. Siam Journal on Multiscale Modeling and Simulation.

[ref-27] Prajit R, Niki P, Ashish V, Irwan B, Anselm L, Jonathon S (2019). Stand-alone self-attention in vision models.

[ref-28] Ronneberger O, Fischer P, Brox T (2015). U-net: convolutional networks for biomedical image segmentation.

[ref-29] Roth S, Black MJ (2005). Fields of experts: a framework for learning image priors.

[ref-30] Roth TPS (2018). Neural nearest neighbors networks.

[ref-31] Tai Y, Yang J, Liu X, Xu C (2017). Memnet: a persistent memory network for image restoration.

[ref-32] Tian C, Xu Y, Li Z, Zuo W, Fei L, Liu H (2020). Attention-guided CNN for image denoising. Neural Networks.

[ref-33] Tian CW, Xu Y, Zuo W (2020). Image denoising using deep CNN with batch renormalization. Neural Networks.

[ref-34] Valsesia D, Fracastoro G, Magli E (2020). Deep graph-convolutional image denoising. IEEE Transactions on Image Processing.

[ref-35] Venkatesh G, Naresh Y, Little S, O’Connor NE (2018). A deep residual architecture for skin lesion segmentation.

[ref-36] Wang Z, Bovik AC, Sheikh HR, Simoncelli EP (2004). Image quality assessment: from error visibility to structural similarity. IEEE Transactions on Image Processing.

[ref-39] Yin X, Zhang Q, Wang H, Ding Z (2020). RBFNN-based minimum entropy filtering for a class of stochastic nonlinear systems. IEEE Transactions on Automatic Control.

[ref-40] Zhang H, Feng L, Zhang X, Yang Y, Li J (2022). Necessary conditions for convergence of CNNs and initialization of convolution kernels. Digital Signal Processing.

[ref-41] Zhang K, Zuo W, Chen Y, Meng D, Zhang L (2016). Beyond a Gaussian denoiser: residual learning of deep CNN for image denoising. IEEE Transactions on Image Processing.

[ref-42] Zhang K, Zuo W, Gu S, Zhang L (2017). Learning deep CNN denoiser prior for image restoration.

[ref-43] Zhang K, Zuo W, Zhang L (2018). FFDNet: toward a fast and flexible solution for CNN based image denoising. IEEE Transactions on Image Processing.

[ref-44] Zhang L, Wu X, Buades A, Li X (2011). Color demosaicking by local directional interpolation and nonlocal adaptive thresholding. Journal of Electronic Imaging.

[ref-45] Zhang Y, Li K, Li K, Zhong B, Fu Y (2019). Residual non-local attention networks for image restoration.

[ref-46] Zhang Y, Tian Y, Kong Y, Zhong B, Fu YR (2021). Residual dense network for image restoration. IEEE Transactions on Pattern Analysis and Machine Intelligence.

[ref-47] Zhang Z, Liu Q, Wang Y (2018). Road extraction by deep residual u-net. IEEE Geoscience and Remote Sensing Letters.

